# No Matter What Images You Share, You Can Probably Be Fingerprinted Anyway

**DOI:** 10.3390/jimaging7020033

**Published:** 2021-02-11

**Authors:** Rahimeh Rouhi, Flavio Bertini, Danilo Montesi

**Affiliations:** 1 Université de Lorraine, CNRS, LORIA, F-54000 Nancy, France; 2 Department of Computer Science and Engineering, University of Bologna, 40126 Bologna, Italy

**Keywords:** camera fingerprint, smartphone identification, user profile linking, digital investigations, social network, classification

## Abstract

The popularity of social networks (SNs), amplified by the ever-increasing use of smartphones, has intensified online cybercrimes. This trend has accelerated digital forensics through SNs. One of the areas that has received lots of attention is camera fingerprinting, through which each smartphone is uniquely characterized. Hence, in this paper, we compare classification-based methods to achieve *smartphone identification* (SI) and *user profile linking* (UPL) within the same or across different SNs, which can provide investigators with significant clues. We validate the proposed methods by two datasets, our dataset and the VISION dataset, both including original and shared images on the SN platforms such as *Google Currents*, *Facebook*, *WhatsApp*, and *Telegram*. The obtained results show that k-medoids achieves the best results compared with k-means, hierarchical approaches, and different models of convolutional neural network (CNN) in the classification of the images. The results show that k-medoids provides the values of F1-measure up to 0.91% for SI and UPL tasks. Moreover, the results prove the effectiveness of the methods which tackle the loss of image details through the compression process on the SNs, even for the images from the same model of smartphones. An important outcome of our work is presenting the *inter-layer UPL* task, which is more desirable in digital investigations as it can link user profiles on different SNs.

## 1. Introduction

In recent years, different social networks (SNs) have revolutionized the web by providing users with specific types of interaction, for instance by sending texts and sharing images and videos. Different SNs meet different needs of users. This means that users are usually active across multiple SNs. It has been reported that on average an Internet user used 8 different SNs at the same time in 2017 [1]. Moreover, many SNs have provided their own dedicated applications for major mobile devices (e.g., smartphones), which has introduced changes in user habits with respect to multimedia content on SNs [2]. In particular, it has led users to take more and more digital images and share them across various SNs [3], making it a challenging task to control the production and propagation of the images and to use the images as digital evidence. From the forensics point of view, the images shared by users on SN platforms could be considered as complementary clues to detect the evidence referenced in a digital crime [4]. In a real scenario, once a digital crime is reported on an SN platform, the police may identify a number of suspects (e.g., friends, relatives and most active users) and collect the electronic devices and the respective profile information on the SNs. With a set of “original images” coming directly from a specific number of the collected devices and the “shared images” taken from suspects’ profiles, *smartphone identification* (SI) and *user profile linking* (UPL) could be achieved. These tasks represent an orthogonal work compared with the work presented in [5] and can provide the police with significant findings and the opportunity to update their dataset to apply to future investigations by creating new fingerprints of the criminals’ smartphones.

More specifically, SI is the task used to identify the source camera generating a given set of images, while UPL is the task used to find the links among the suspects’ profiles. It is worth mentioning that a user would be linked to other profiles even if there is not a direct friendship between the profiles on the same or different SN platforms. In recent years, methods based on camera sensor imperfections have been known as a robust approach for smartphone fingerprinting applied to digital investigations due to their stability against environmental conditions [6,7,8]. The photo-response non-uniformity (PRNU) approach is most suitable for defining the pattern noise (PN) of camera sensor imperfections [9,10]. The PN can be approximated as the average of residual noises (RNs) present in each image captured by the camera. The RN is estimated as the difference between the image content and its denoised version obtained through a denoising filter [10]. Due to the effectiveness of PRNU, in this paper, we take advantage of PRNU in the classification of both “original” (or native) and “shared images” within a set of investigated profiles on SNs to achieve SI and UPL.

### 1.1. Problem Statement

**Figure 1:**
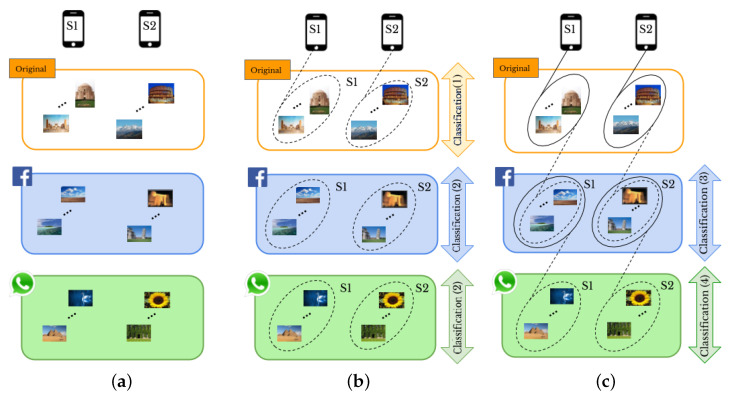
A visual example of the proposed methods: (**a**) domain of the problem, (**b**,**c**) classification-based approaches for smartphone identification (SI) and user profile linking (UPL) by “original” and “shared images”, respectively. The labels (1) to (4) refer to Figure 2 presenting all the combinations of “original” and “shared images”.

Given a set of images, “original” or “shared images”, taken by a given number of smartphones, and a set of user profiles, as shown in Figure 1a, we aim to perform SI and UPL tasks based on classification of smartphones’ camera fingerprints. In particular, a visual example of the proposed methods for two smartphones and two SNs, *Facebook* and *WhatsApp*, is provided in Figure 1b,c. For SI, we consider the following cases:

1.1 *Original-by-original SI* is the task used to detect the source cameras from which a set of “original images” directly coming from smartphones have been taken, see the arrow labeled “Classification (1)” in Figure 1b.
1.2 *Social-by-original SI* represents the task used to identify the source cameras of a given set of “shared images”, see the arrow labeled “Classification (3)” in Figure 1c. In this case, the “original images” are input data and allow one to define the smartphone camera fingerprints.

Moreover, the UPL task is categorized into two cases: within the same SN and across different SNs, resulting in the following:

2.1 *Intra-layer UPL* is the task used to link a given set of user profiles within the same SN using “shared images”, see the arrows labeled “Classification (2)” on *Facebook* and *WhatsApp* in Figure 1b. Through this task, the profiles that share images from the same source are linked within the same SNs.
2.2 *Inter-layer UPL* represents the task used to link a set of user profiles across different SNs by using “shared images”, see the arrow labeled “Classification (4)” in Figure 1c. Through this task, the profiles from different SNs that share images from the same source are linked.

### 1.2. Contribution

**Figure 2:**
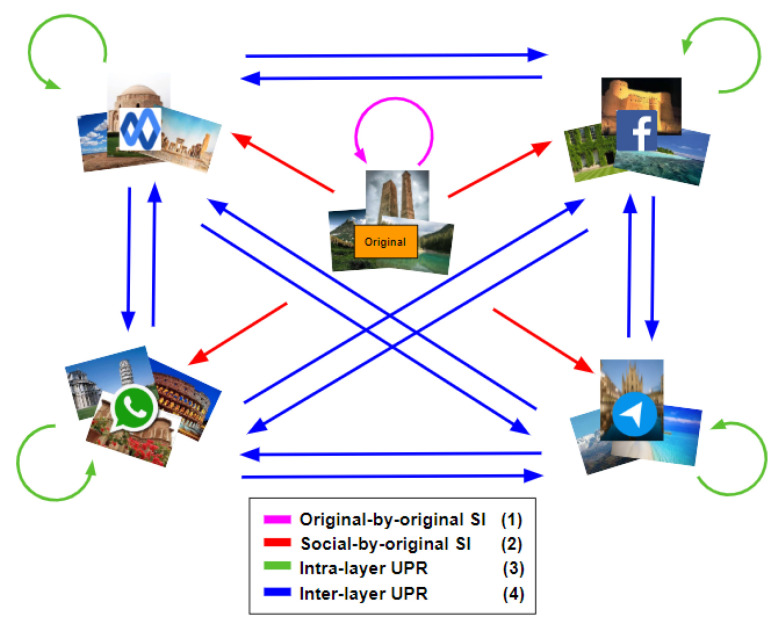
All the possible combinations of “original” and “shared images” in the proposed methods. The green and magenta rounded arrows from *A* to *A* imply classifying images of *A*, while the blue and red straight arrows from *A* to *B* mean that we use the classified images of *A* to classify the images of *B*.

In this paper, we apply both “original” and “shared images” to fingerprint smartphones. We assume that the number of smartphones is known. Figure 2 shows all the combinations of both types of images. Labels (1)–(4) make a connection with Figure 1b,c, presenting the same meaning. We investigate different approaches, such as pre-trained CNN and clustering methods, for *original-by-original SI* and *intra-layer UPL* tasks, and we apply a neural network model for the *social-by-original SI* and *inter-layer UPL* tasks. According to the comparison results (see Section 4.1 and Section 4.2 for more details), k-medoids technique [11] effectively classifies “original” and “shared images” and achieves *original-by-original SI* and *intra-layer UPL* (i.e., the green and magenta rounded arrows in Figure 2). In addition, a classification approach based on artificial neural networks (ANNs) effectively achieves *social-by-original SI* and *inter-layer UPL* (i.e., the blue and red straight arrows in Figure 2). In particular, we classify the “shared images” by exploiting the fingerprints derived from the obtained classes, refer to Section 3.4 for more details.

Analyzing a huge number of images on all SN platforms is an unfeasible task; for this reason, in a real-world scenario investigators identify a restricted number of suspects and collect the relative devices and user profile information. Accordingly, to evaluate the proposed methods, we use our real dataset that consists of 4500 images captured by 18 different smartphones. The dataset was uploaded and downloaded on 4 of the most popular SNs, namely *Google Currents* (Google+ was discontinued in April 2019 and enterprise accounts were transitioned to Google Currents ), *Facebook*, *WhatsApp*, and *Telegram*. In addition, we validate our proposed methods by the VISION image dataset [12]. The obtained results show the effectiveness of the proposed methods, even for the images degraded through the compression process on the applied SNs. Moreover, the methods are device-independent and able to distinguish the same model of smartphones. An important result of our work is applying the *inter-layer UPL* task to link a given set of user profiles on different SN platforms. This is more desirable in digital investigations because on average, users are active on multiple SNs [1].

The rest of the paper is organized as follows. Section 2 provides a summary of the SI and UPL tasks proposed in the literature. In Section 3, we describe the proposed methods. Experiments and their results are discussed in Section 4. In Section 5, limitation and significance of the proposed methods are presented. Some concluding remarks are made in Section 6.

## 2. Related Works

Smartphones have several built-in sensors that measure motion, orientation, and various environmental conditions. All of these components present hardware imperfections created during the manufacturing process that uniquely characterize each smartphone. The smartphone fingerprint formed by these imperfections has been known as a reliable characteristic making a smartphone trackable [7,8,9].

A lot of attempts have been made to get smartphone fingerprints using a variety of sensors such as accelerometers [13], gyroscopes [14], magnetometers [15,16], cameras [17], and paired microphones and speakers [18]. The camera could be considered a built-in sensor that is less invasive and more suitable for source camera identification [6]. A pioneering work [9] introduced the PRNU technique to obtain camera sensor noise. A significant advantage of the PRNU is that it remains stable under different environments. In addition, it is considered a reliable fingerprint that efficiently characterizes the digital device that generated the image [19,20].

Most of the works proposed for SI and smartphone image classification were implemented on the “original images”, e.g., [20,21,22,23]. However, identification by “shared images” is challenging because of the images’ compression. Only a few works, e.g., [12,24,25], applied shared images or videos from, for example, *Facebook*, *YouTube*, and *Twitter*. All the mentioned works used “shared images” for only SI not UPL.

Different approaches have been proposed for the UPL task. For example, [26] exploited user activities on SNs. They collected logs filed within the device through a manual investigation and used them to match user profiles. Their experiments showed that the method failed for BlackBerry devices. Similarly, reference [2] monitored user activities and collected a variety of artifacts, such as usernames, passwords, login information, personal information, uploaded posts, and exchanged messages. All of this information was gathered for the digital investigations. The authors of [27] used the Jaro–Winkler distance algorithm [28], to compare the account information of users, such as username, friends, and interests, from accounts on different SNs for profile matching. Iofciu et al. [29] introduced a method based on the combination of user IDs and tags to recognize users through the social tagging system.

The works of [30,31] presented a framework for UPL across SNs considering profile attributes. The framework assigns a different similarity measure to each attribute. The authors of [32] introduced a method that was not dependent on login credentials. The behavioral traits of users were applied to link users. Zafarani et al. [33] applied behavioral patterns to establish a mapping among identities of individuals across social media sites. The authors of [34] used datasets such as call records and matched the obtained histograms of users’ data representing their fingerprints to identify users. In [35], user activities on SNs were analyzed to find trust interactions between the users. However, there are still some problems with these approaches. The information of users’ identities could be diverse on different SNs [36]. The users may select different nicknames and E-mail addresses, resulting in incorrect matching between the real person and the accounts [33].

Hence, in this paper, we use a different approach based on supervised and unsupervised classification techniques, extending our previous works [37,38,39]. What makes our work innovative is using images from one SN to identify smartphones applied on another SN, which provides user profile linking across different SN platforms. In addition, we apply our proposed methods to larger datasets covering images from different or even identical models of smartphones.

## 3. Methodology

We first provide a brief background on RN extraction and PN computation, namely smartphone camera fingerprinting. Then, we describe the pre-processing phase that enables definition of several parameters, such as the orientation, size, and channel of the images. Finally, we explain SI and UPL across SNs based on classification techniques. To evaluate our methods, we gathered a dataset including 4500 images from 18 different smartphones. Through the paper, we call our dataset “Lab Dataset”, i.e., $D_{L}$. Based on our previous work [40], the minimum number of images per samrtphone to get a reliable fingerprint is 50. Hence, for each smartphone, we collected 250 images. Then, we kept a subset of 50 “original images” (O) and uploaded and downloaded 50 images on each of the four selected SNs: *Google Currents* (G), *WhatsApp* (W), *Facebook High Resolution* (FH), and *Telegram* (T). Correspondingly, we have the datasets $D_{L}^{O}$, $D_{L}^{G}$, $D_{L}^{W}$, $D_{L}^{\mathrm{FH}}$, and $D_{L}^{T}$. The characteristics of the applied smartphones in $D_{L}$ are shown in Table 1. We use also the VISON image dataset including a different number of images taken by 35 smartphones. The images are divided into *flat*, which is a set of images of walls and skies, and *generic*, which is a set of images without limitations on orientation or scenario. The images were shared through WhatsApp and Facebook (in both high and low resolutions). We use only generic images in our experiments. We call the datasets $D_{V}^{O}$, $D_{V}^{W}$, $D_{V}^{\mathrm{FH}}$, and $D_{V}^{\mathrm{FL}}$ corresponding to the O, W, FH, and *Facebook Low Resolution* (FL) images. The lowest and the highest resolutions of images for each SN in the datasets $D_{L}$ and $D_{V}$ are presented in Table 2.

### 3.1. Smartphone Fingerprinting

We use the PRNU approach, proposed by [41], to extract the RN left by sensor imperfections in each image. Let *I* and d() be, respectively, an image and a denoising filter. The RN is computed as follows:

(1) RN=I−d(I)

Then, the PN (i.e., the smartphone camera fingerprint) is approximated by averaging the RNs of *n* images of camera *k* as follows:

(2) $$PN_{k}=\frac{1}{n}\sum_{j=1}^{n}RN_{j}$$

According to (1) and (2), *n* and d() are the two main factors that affect the quality of the PN. In particular, the more images taken by a certain source are provided, the higher the quality of PN is acquired [42]. We use Block-matching and 3D filtering (BM3D) as the denoising filter d(). It has shown promising effectiveness regarding the *peak signal-to-noise ratio* and visual quality, even for high levels of noise and scaled images [7,43,44].

### 3.2. Pre-Processing

The collected images come from different smartphones with different characteristics, such as orientation and size. We do the pre-processing phase to make a coordination between images in terms of orientation, size and channel. The aim is to balance a trade-off between the computational cost and the effectiveness of the proposed methods.

The image orientation depends on the rotation of the acquisition smartphone, which could be either portrait or landscape. Smartphone fingerprinting based on camera sensors is dependent on the orientation of images. Accordingly, the orientation has to be normalized for all the applied images. Although for “original images”, the metadata, which are available through Exchangeable Image File Format (EXIF) [45], could be a solution to obtain the right orientation, this is not applicable to “shared images”. The reason is that the SN platforms usually remove the metadata, such as orientation, during the uploading and downloading of the images. Hence, for the “shared images”, we only align the images to either portrait or landscape orientation based on the spatial resolution [12]. This may not entirely resolve the orientation problem and affects the classification, but it can be alleviated.

In our previous work [38], in fingerprinting smartphones, we tested different channels of images, i.e., R, G, and B in RGB color space, and Y in YCbCr color space, among which the Y channel led to the best results. Therefore, we use the Y channel (gray-scale version) of images in this paper.

Unlike most of the presented works in the literature, which mostly cropped the central block of the extracted RNs, we use resizing. Generally, resizing the images involves up-scaling or down-scaling the images to a specific resolution. After extracting the RNs from gray-scale images, the obtained RNs are resized to an optimal size based on bicubic interpolation [46]. We will present some experiments in Section 4 to show the impact of resizing compared with cropping RNs.

### 3.3. Original-By-Original Smartphone Identification and Intra-Layer User Profile Linking

We apply supervised and unsupervised classification to images (see Figure 1b). More specifically, we do supervised classification using different pre-trained convolutional neural networks (CNN) such as GoogleNet [47], SqueezeNet [48], Densenet201 [49], and Mobilenetv2 [50]. In particular, we have added a convolutional layer to adapt the size of the images to the network input, retaining the weights from the previous training on the ImageNet dataset. As an unsupervised classification, we use k-means, k-medoids, and hierarchical techniques, which are performed based on a similarity measure in such a way that the objects in the same class have more similarity compared with those in different classes [51]. In the hierarchical classification technique, the objects are typically organized into a dendrogram (tree structure), where leaf nodes represent the individual data and the root is the whole dataset. The middle nodes show merged groups of similar objects [52]. In partitional classification such as k-means [53], and k-medoids the objects are divided into some partitions, each of which is considered as a group. The partitional classification starts by initializing a set of *k* class centers. Then, each object is assigned to the class whose center is the nearest [11,54]. K-medoids is an expensive approach, but it is a more reliable technique in the presence of noise and outliers compared to the other unsupervised classification methods [55].

**Figure 3:**
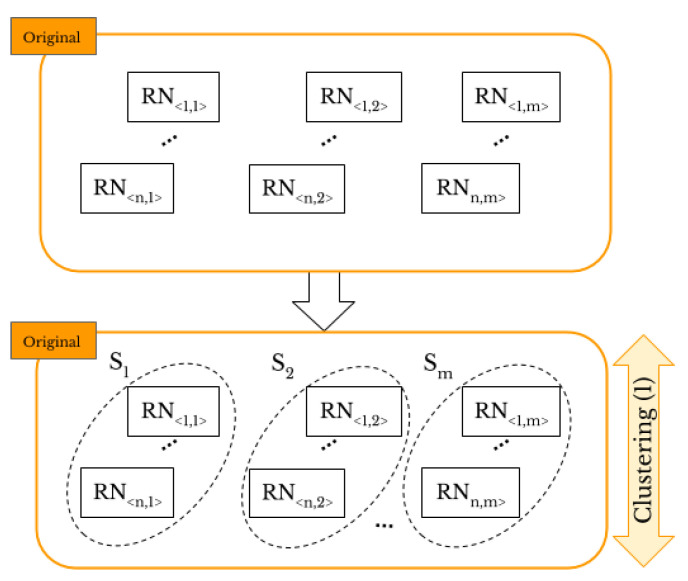
*Original-by-original SI*: the “original images” are classified according to the smartphone’s source camera.

We compare the CNN, hierarchical, k-means, and k-medoids techniques to classify the “original images” and achieve *original-by-original SI* and select the best technique for classification of smartphone camera fingerprints. Then, in a similar way, we classify the “shared images” to achieve *intra-layer UPL*. Figure 3 shows the task of *original-by-original SI*. Through the proposed methods, the number of smartphones under investigation has to be provided. Let *I* be a set of the “original images”, and $S=\{S_{1},S_{2},\ldots ,S_{m}\}$ be a set of *m* camera sources. We aim to classify the images of *I* into the right sources of *S*, where each camera source $S_{i}$ has its own set of images, that is $I_{\langle 1,i\rangle },\ldots ,I_{\langle j,i\rangle },\ldots ,I_{\langle n,i\rangle }\in S_{i}$. Thus, we have the full dataset $I=⋃I_{\langle i,j\rangle },\;\forall \;i=1,\ldots ,n$ and j=1,…,m, where *n* is the number of the collected images for each of the *m* smartphones. Firstly, we extract the RNs of the “original images” such that $RN_{<i,j>}$ is the RN corresponding to ith image taken by jth smartphone. Then, we use correlation as the similarity measure because it is the optimal metric for multiplicative signals such as PRNU [41]. The correlation between $RN_{\langle a,b\rangle }=\left[x_{1},\ldots ,x_{l}\right]$ from $I_{\langle a,b\rangle }$ and $RN_{\langle c,d\rangle }=\left[y_{1},\ldots ,y_{l}\right]$ from $I_{\langle c,d\rangle }$, such that *l* is the total number of pixels forming images $I_{\langle a,b\rangle }$, $I_{\langle c,d\rangle }$ and the two related RN vectors, is defined as follows:

(3) $$\rho =\frac{\sum_{i=1}^{l}(x_{i}-{\bar{RN}}_{\langle a,b\rangle })\left(y_{i}-{\bar{RN}}_{\langle c,d\rangle }\right)}{\sqrt{\sum_{i=1}^{l}(x_{i}-{\bar{RN}}_{\langle a,b\rangle }{)}^{2}\sum_{i=1}^{l}(y_{i}-{\bar{RN}}_{\langle c,d\rangle }{)}^{2}}}$$

where ${\bar{RN}}_{\langle a,b\rangle }$ and ${\bar{RN}}_{\langle c,d\rangle }$ represent the means of the two RN vectors. We create a matrix ζ containing correlations between each pair of the extracted RNs. As a result of the varying qualities of PNs of different cameras, the average correlation between the RNs from one camera may differ from that of other camera [20]. This problem makes the classification of PNs more challenging. To address this problem, an alternative similarity measure is calculated based on shared κ-nearest neighbors (SNN) proposed by [56]:

(4) $$W\left(d_{i},d_{j}\right)=\left|N\left(\rho_{i}\right)\cap N\left(\rho_{j}\right)\right|$$

where $\rho_{i}$ and $\rho_{j}$ are two elements in the correlation matrix ζ, and $N(\rho_{i})$ and $N(\rho_{j})$ are the SNN of $\rho_{i}$ and $\rho_{j}$, so $W(\rho_{i},\rho_{j})$ results in the number of κ-nearest neighbours shared by $\rho_{i}$ and $\rho_{j}$. Then, we apply classification to the resulted matrix W from SNN.

**Figure 4:**
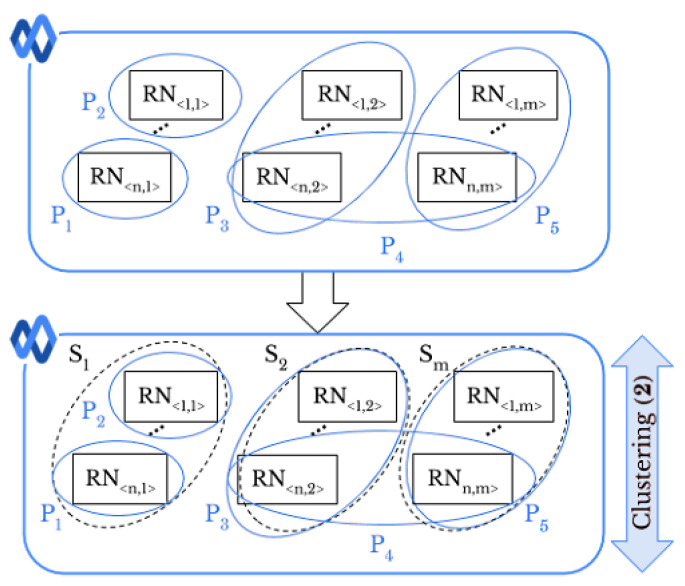
*Intra-layer UPL* task: profiles $P_{1}$ and $P_{2}$ are linked since they share images taken from the same smartphone $S_{1}$.

Smartphone identification deals with 1-to-m matching problem and determines which smartphone out of *m* took a given image. Therefore, the stopping criterion in hierarchical classification and the parameter *k* in k-means and k-medoids are set to the number of smartphones, i.e., m=18 and m=35 for $D_{L}$ and $D_{V}$, respectively. The number of smartphones represents the number of classes as the output of various networks used. All the classification approaches associate each RN with a label that represents the related source of the image. Similarly, we address the *intra-layer UPL* task, as shown in Figure 4.

Let $D^{\;x}$ be a set of images where x∈{G,W,FH,FL, T}. Each image in $D^{\;x}$ has a specific profile tag $P_{i}$ that represents the ith user’s profile on the SN *x* the image comes from. Like *original-by-original SI*, we exploit the full pairwise correlation matrix of the extracted RNs to classify $D^{\;x}$ images into the right sources of *S*. Then, by using the resulted classes and profile tags, we are able to link profiles. Moreover, we can determine whether a user uploaded images taken by one or more smartphones. In the first case, if within two different profiles there are images that are in the same class $S_{i}$, these profiles could be linked. For instance, in Figure 4, identification of smartphone $S_{1}$ leads to a matching between the profiles $P_{1}$ and $P_{2}$. In the second case, if the images belonging to the same profile are grouped in different classes, it means that the user uploaded the images from different smartphones. In Figure 4, the user of profile $P_{4}$ has shared images taken by two different smartphones, namely $S_{2}$ and $S_{m}$.

### 3.4. Social-By-Original Smartphone Identification and Inter-Layer User Profile Linking

**Figure 5:**
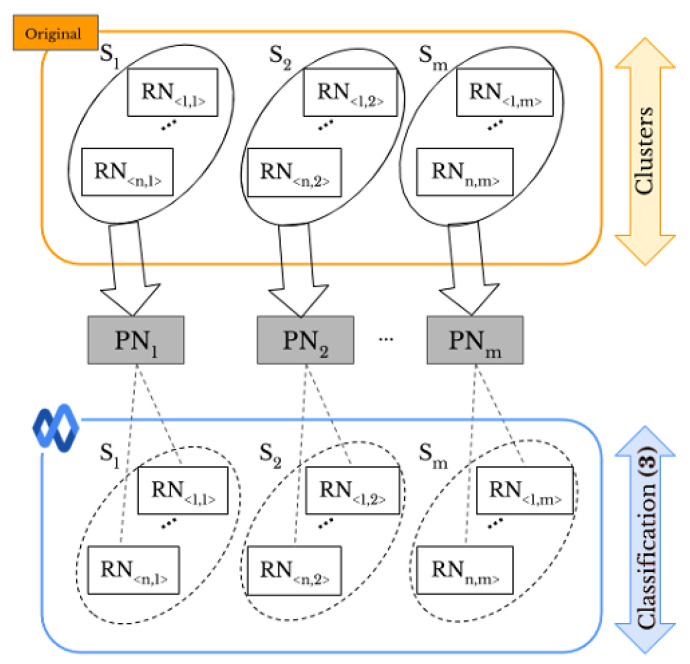
*Social-by-original SI* task based on classification approach: the classified “original images” are used to train the ANN and classify the “shared images”.

Here, we exploit the obtained classes, from *original-by-original SI* and *intra-layer UPL* tasks, as ground truths of the fingerprinted smartphones to classify “original” or “shared images” into *m* classes. Generally, ANNs, inspired by the biological form of the human neural system, have proven their effectiveness in classification tasks [57]. They are very flexible in learning features and can solve non-linear problems. Compared with the other classifiers such as support vector machine, extreme learning machine, and random forest, ANNs are more fault tolerant [58]. As a mathematical model, an ANN consists of a set of attached neurons called processing units. Neurons are organized in layers. The output of a neuron is stated as f(h), where f() is the *activation function*, and *h* is computed as follows:

(5) $$h=\sum_{i=1}^{s}w_{i}x_{i}+b$$

where $x_{i}$ and $w_{i}$ are the input data and weight of the neuron, respectively; *b* is the bias; and *s* is the total number of input connections of the neuron [59]. For a desirable classification, the weights of the ANN should be tuned. This process is called *training* or *learning* [60]. A *multi-layer perceptron* (MLP) is a kind of ANN composed of one or several hidden layers of neurons [61]. An MLP is trained by using a *back propagation* (BP) algorithm such that it minimizes the *mean squared error* (MSE), which is formulated by:

(6) $$\mathrm{MSE}=\frac{1}{N}\sum_{i=1}^{N}{\left(T_{i}-O_{i}\right)}^{2}$$

where *O* and *T* are matrices representing the labels predicted by ANN and the class labels of the inputs, respectively, and *N* is the number of samples. We will use the classified images that are the outcome of the previous task and ANN to perform both *social-by-original SI* and *inter-layer UPL*. The *social-by-original SI* task is shown in Figure 5. We first define the fingerprint $PN_{i}$ corresponding to the obtained classes from the set *I*, such that $PN_{i}$ transitively identifies the smartphone $S_{i}$. Then, by (3), we calculate the correlation values between each pair of RNs extracted from the images in $D^{\;x}$, and the obtained PNs. For example, a correlation matrix of the size 900×18 is formed corresponding to 900 RNs in $D_{L}^{G}$ to be classified according to 18 smartphones in $D_{L}^{O}$ which have already been identified in the classification. The matrix is used for training and test the ANN through a 10-fold cross-validation model [62]. In particular, in every 10 iterations, the ANN is given 90% of the rows in the correlation matrix and corresponding class labels (smartphone labels by which the RNs in $D_{L}^{G}$ were generated) as the ground truth. In the test, the trained ANN is provided by 10% of the rows in the correlation matrix to classify each image in $D_{L}^{G}$, called *social-by-original SI*. By using the 10-fold approach, all the samples in the correlation matrix are tested as there is a swap between training and test in each iteration.

**Figure 6:**
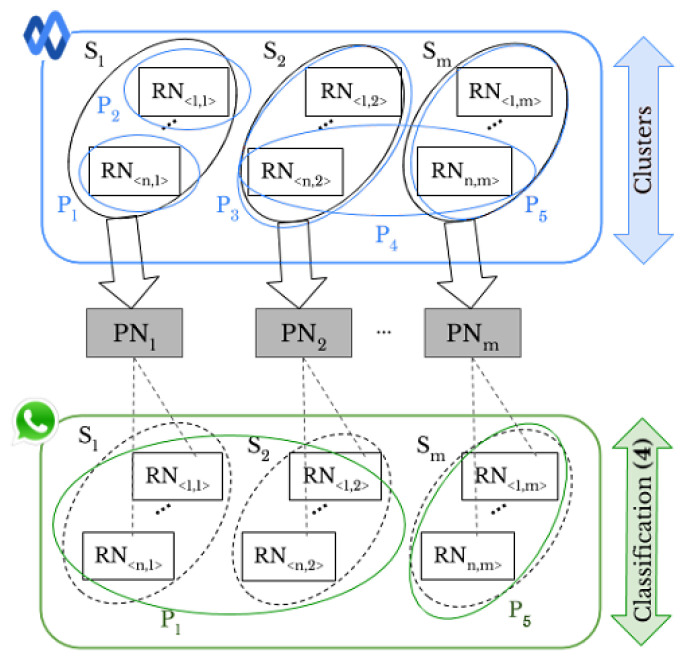
*Inter-layer UPL* task based on classification approach: to classify the “shared images” on a given social network (SN) (e.g., *WhatsApp*), the ANN is trained by using the obtained classes of “shared images” on a different SN (e.g., *Google Currents*).

In *inter-layer UPL* task, as shown in Figure 6, the profile tag $P_{i}$, where *i* represents the *i*th profile on a given SN, allows one to link user profiles across different SNs. The $PN_{i}$ is defined by using the classes obtained from *Google Currents*, and the ANN is trained to classify the *WhatsApp* images. After the classification, the profile $P_{1}$ on *WhatsApp* is linked to the profiles $P_{1}$, $P_{2}$, and $P_{3}$ on *Google Currents* because they share images taken from the same smartphones $S_{1}$ and $S_{2}$. Similarly, the profile $P_{5}$ on *WhatsApp* is linked to the profiles $P_{4}$ and $P_{5}$ on *Google Currents*.

We tested different topologies for the applied ANNs in terms of training method, activation function, and the number of hidden layers. As a result, an appropriate effectiveness of *social-by-original SI* and *inter-layer UPL* is achieved by the simple ANN’s architecture shown in Table 3. In particular, we use *trainscg* as the training function that updates weight and bias values based on the *scaled conjugate gradient training* algorithm, and the *logistic sigmoid* as activation function that provides an appropriate convergence in the training. In particular, the applied activation function is defined as follows:

(7) $$f\left(h\right)=\frac{1}{1+e^{-h}}$$

where *h* is obtained by (5).

## 4. Experimental Results

In this section, the results of SI and UPL are presented. In particular, the results of *original-by-original SI*, *social-by-original SI*, *intra-layer UPL*, and *inter-layer UPL* are provided, respectively. The proposed methods were implemented in MATLAB, version R2019a on a laptop with the following characteristics: Intel Core i7-6500U (2.93 GHz), 16 GB of RAM, and Windows 10 operating system. In each of these tests, to evaluate the classification processes, we calculate several measures. Let TP be a set of images to which the method has correctly assigned class labels, while that it has correctly not assigned is represented by TN. In addition, FP is the set of images to which the method has wrongly assigned class labels and FN is the set of images that the method has wrongly not assigned. Accordingly, Sensitivity (SE), Specificity (SP), Rand Index (RI), Adjusted Rand Index (ARI), F1-measure (F), and Purity (P) are defined as follows:

(8) $$\mathrm{SE}=\frac{\left|\mathrm{TP}\right|}{\left|\mathrm{TP}|+|\mathrm{FN}\right|}$$

(9) $$\mathrm{SP}=\frac{\left|\mathrm{TN}\right|}{\left|\mathrm{TN}|+|\mathrm{FP}\right|}$$

(10) $$\mathrm{RI}=\frac{\left|\mathrm{TP}|+|\mathrm{TN}\right|}{\left|\mathrm{TP}|+|\mathrm{FP}|+|\mathrm{TN}|+|\mathrm{FN}\right|}$$

where |.| denotes cardinality of the related set, i.e., TruePositive(TP), TrueNegative(TN), FalsePositive(FP), or FalseNegative(FN). The value of RI varies between 0 and 1, respectively showing no agreement and full agreement between the classification results and the ground truth. For two random classes, the average of RI, i.e., $\bar{\mathrm{RI}}$ is a non-zero value. To get rid of this bias, ARI was proposed by [63]:

(11) $$\mathrm{ARI}=\frac{\mathrm{RI}-\bar{\mathrm{RI}}}{1-\bar{\mathrm{RI}}}$$

(12) $$F=\frac{2.|\mathrm{TP}|}{2.|\mathrm{TP}|+|\mathrm{FP}|+|\mathrm{FN}|}$$

(13) $$P=\frac{\sum_{i=1}^{\left|C\right|}\frac{|{\hat{c}}_{i}|}{|c_{i}|}}{\left|C\right|}$$

where $C=\{c_{1},c_{2},\ldots ,c_{m}\}$ is the set of the obtained classes corresponding to *m* smartphones in dataset, $\hat{c_{i}}$ denotes the number of RNs with the dominant class label in the class $c_{i}$, and $|c_{i}|$ is the total number of RNs in $c_{i}$.

As described before, we evaluate the effectiveness of the ANN in the training phase as well as its generalization capability by using 10-fold cross-validation. Firstly, a matrix including the correlations between the extracted RNs and the obtained PNs are calculated based on (3). The *i*th row of the matrix includes the similarities between the *i*th RN and all the resulted PNs from the classification. The rows related to the same smartphone are shuffled to have an order-independent evaluation. Then, they are divided into 10 folds so that each of them includes an equal number of samples for each smartphone. In each of 10 iterations of the cross-validation, nine folds and one independent fold are used respectively for “training set” and “test set”. For example, in $D_{L}^{O}$ we have 50 images for each smartphone, so we use 850 and 50 rows, respectively, in training and test at each iteration. The 10-fold cross-validation process is repeated 10 times, and finally, the average values obtained from the measures in (8)–(13) are considered as the ANN results.

### 4.1. Original-By-Original Smartphone Identification Results

In this experiment, we use “original images” to identify their acquisition smartphones, which is called the *original-by-original SI* task. As shown in Table 1, these images have a high resolution, so the results can be considered as a benchmark for the capability of the classification in the best case. Furthermore, we exploit this experiment to perform some pre-processing in terms of size for all the applied images in the datasets. In particular, in the pre-processing phase, we use the k-medoids method because it is a more reliable technique in the presence of noise and outliers.

**Figure 7:**
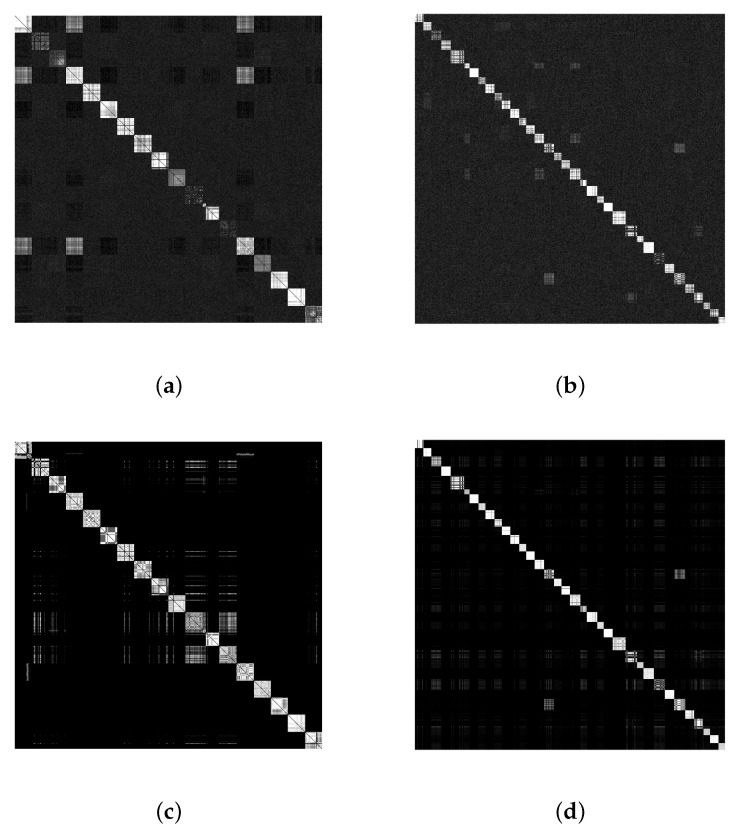
Pairwise similarities of residual noises (RNs): (**a**,**b**) without and (**c**,**d**) with using shared κ-nearest neighbor, respectively from left to right for $D_{L}^{O}$, κ = 20, and $D_{V}^{O}$, κ = 70.

Based on Table 4, to obtain the optimal resolution in resizing, we resize the extracted RNs form the images in $D_{L}^{O}$ with different resolutions, i.e., 128 × 128, 256 × 256, 512 × 512, 960 × 544, 1024 × 1024, 1280 × 1024, and 1536 × 1536. Then, we do classification by k-medoids. We choose the size of 1024×1024 as it results in the highest values of all the measures, i.e., SE, SP, RI, ARI, F, and P compared with the other resolutions. In addition, Figure 7 shows the impact of SNN on the pairwise correlation matrices of the datasets $D_{L}^{O}$ and $D_{V}^{O}$. Comparing the subfigures (c) and (d) with (a) and (b), it can be seen that the average of intra-camera correlations, i.e., the diagonal parts, has increased while the average of the inter-camera correlations has decreased. This improvement in the correlations between RNs produces better results for k-medoids. The value of κ in SNN for each dataset was experimentally determined. Different values were tested and κ=20 and κ=70 generated the best results in the classification for $D_{L}^{O}$ and $D_{V}^{O}$, respectively.

**Figure 8:**
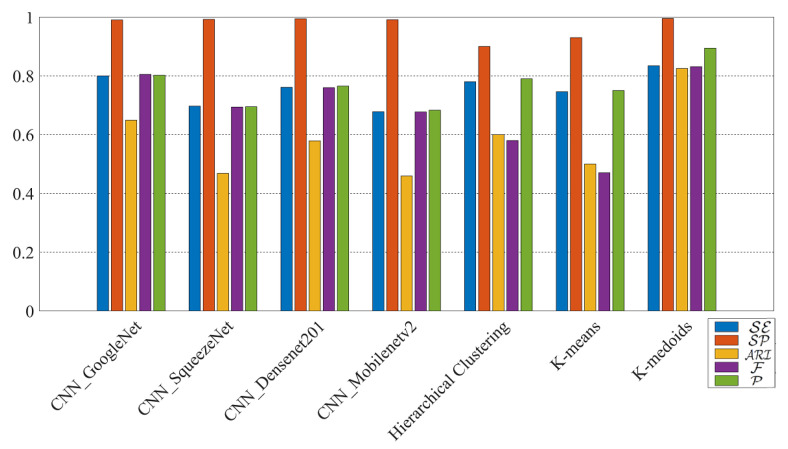
Results (%) of original-by-original SI by using different methods on $D_{V}^{O}$ with the RN resolution 1024×1024.

The comparison results among CNN models, hierarchical clustering, k-means, and k-medoids techniques applied to $D_{V}^{O}$ are shown in Figure 8. The results confirm that k-medoids is the best to classify RNs, even for RNs extracted from images from identical models of smartphones. According to Table 5, the results of k-medoids on both the datasets $D_{L}^{O}$ and $D_{V}^{O}$ show the effectiveness of the classification with the resolution 1024×1024.

### 4.2. Social-By-Original Smartphone Identification Results

In this test, we use both “original” and “shared images” to present *social-by-original SI*. Firstly, we exploit *Google Currents* images in $D_{L}$ to set up the architecture of the applied ANNs as *Google Currents* images provide the highest resolution. Accordingly, the test could also be considered as a benchmark for the ANNs used for the other SNs. In particular, to tune the number of neurons in the hidden layer, we consider the classes of the “original images” from the previous test and classify the *Google Currents* images.

**Figure 9:**
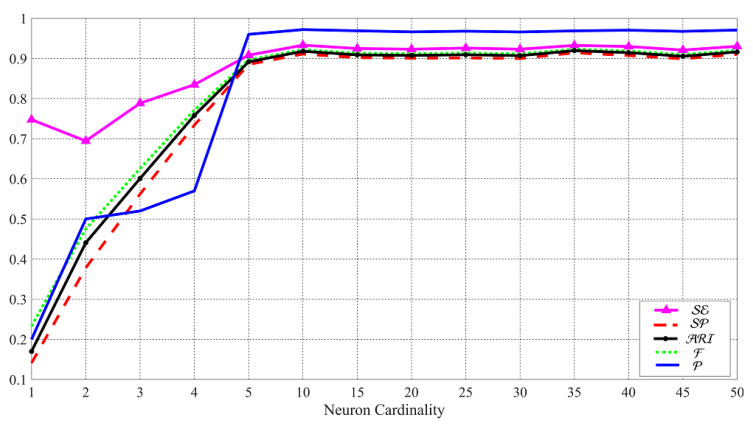
Results (%) of *social-by-original SI* for systematically increasing the number of neurons in the hidden layer of ANN. The images in $D_{L}^{G}$ are classified by the obtained classes of images in $D_{L}^{O}$ and the trained ANN.

Based on Figure 9, by systematically increasing the number of neurons, the classification results are improved in terms of SE, SP, ARI, F and P. Although the highest values are resulted in the cardinality of 35, up to the cardinality of 50, there are still some fluctuations in the values. For this reason, we set the number of the neurons to 50 in our experiments. The tuning phase of the ANNs can also be used as a benchmark for the capability of the classification in the best case because the “original images” and *Google Currents* images have the highest resolution in the dataset.

The results of *social-by-original SI* for both datasets $D_{L}$ and $D_{V}$ are shown in Table 6. The *social-by-original SI* enables identification of smartphones in spite of the fact that the pictures get downgraded during the uploading and downloading process.

### 4.3. Intra-Layer User Profile Linking Results

In this section, we discuss the results of *intra-layer UPL*. In particular, this test exploits “shared images” to determine whether a given set of user profiles within the same SN are linked. Table 7 shows the results on the “shared images” in both $D_{L}$ and $D_{V}$. The best results are related to $D_{L}^{G}$. The reason is that *Google Currents* images have the same resolution as the “original images” confirming that the compression algorithm on this SN results in less elimination of image details, (see Table 2). Although the other SNs compress the images more than *Google Currents*, the method has returned good results confirming the effectiveness of the method in the task of *intra-layer UPL*.

### 4.4. Inter-Layer User Profile Linking Results

This last test is the most challenging. We demonstrate that the proposed method is able to link a restricted set of user profiles across different SNs. In other words, we verify whether two sets of images from different user profiles on different SNs are linked, that is *inter-layer UPL*. The strengths of our method include the possibility to exploit images from different SNs, not only the “original images”, but also the robustness in spite of the fact that some SNs degrade the resolution of the images more than others. We consider all the different combinations of the selected SNs for each dataset, as shown in Figure 2. The results for all the possible pairs of SNs are presented in Table 8 and Table 9.

It is worth mentioning that the images in $D_{L}$ used for experiments of *inter-layer UPL* on different SNs are not from the same scenes, making a more similar real-life situation. Among the results in Table 8, using *Google Currents* images to classify the images on the other SN datasets, i.e., $D_{L}^{W}$, $D_{L}^{\mathrm{FH}}$, and $D_{L}^{T}$ produce the highest values of SE, SP, ARI, F, and P, as shown in the first rows of Table 8. For $D_{V}$, using images in $D_{V}^{W}$ to classify the images in the other datasets, i.e., $D_{L}^{\mathrm{FH}}$ and $D_{L}^{\mathrm{FL}}$ concluded the best results. It is interesting that the classification of the images in $D_{L}^{\mathrm{FL}}$ in inter-layer UPL compared with the classification of the images in intra-layer UPL generates better results. Given the results, it is proven that the proposed methods are reliable enough to match user profiles on the selected SNs.

## 5. Discussion

We have presented *smartphone identification* (SI) and *user profile linking* (UPL). Analyzing a huge number of images on all SN platforms is an unfeasible task. In addition, the digital investigation is operated on a restricted set of devices, suspects’ profiles, and a given set of investigated images. Hence, we considered a scenario in which the number of smartphones has to be provided. Although in some applications it is not and clustering is used instead [5,23], applying classification is preferable which provides more accurate results compared with clustering.

Based on our work, it can be implied that despite the advances in deep learning techniques in classification with different CNN models, traditional techniques like k-medoids can still achieve high performing smartphone image classification tasks. K-medoids only needs one parameter to be set that is the number of smartphones in our application, while for CNN models lots of parameters have to be set which makes the classification more challenging and computationally expensive.

An important outcome of our work is presenting the *inter-layer UPL* task, which is more desirable in digital investigations as it links user profiles on different SNs. The proposed methods in the combination of the other types of information such as GPS, users’ E-mail addresses, and login information can also help for user profile linking.

## 6. Conclusions

In this paper, we have compared different classification methods to achieve SI and UPL. The methods can help forensic investigators to find significant information from digital crimes when a set of images captured by a specific number of smartphones and shared on a set of investigated user profiles are provided. We have evaluated our methods on different datasets, i.e., our dataset and VISON dataset. The obtained results show that with an acceptable error margin, k-medoids achieves the best results compared with k-means, hierarchical approaches, and different models of convolutional neural network (CNN) in the classification of the images. In particular, the results indicate that even in the worst case k-medoids can provide the values of F1-measure 75% and 77%, for SI and UPL tasks, respectively. The results confirm the effectiveness of the methods, even with the same models of smartphones. The methods are applicable to images compressed on SNs, and there is no need to hack the user’s smartphone for fingerprinting. An important outcome of our work is presenting the *inter-layer UPL* task, which is more desirable in digital investigations because it links user profiles on different SNs. The methods will become even more powerful when considering other types of information such as GPS, users’ E-mail addresses, and login information, to name a few. Through the proposed methods, the number of smartphones under investigation has to be provided. However, in our future work, we plan to present an algorithm to classify all shared images on the suspect’s profile, without prior knowledge of the source cameras. In addition, the relationship between the two parameters of the number of smartphones and the number of images needed per smartphone can be investigated to handle the uncertainty of these two parameters.

## Figures and Tables

**Table 1:** Characteristics of smartphones in $D_{L}$.

Phone ID	Brand	Model	Resolution
S1	LG	Nexus 4	3264×2448
S2	Samsung	Galaxy S2	3264×2448
S3	Apple	iPhone 6+	3264×2448
S4	LG	Nexus 5	3264×2448
S5	Huawei	Y550	2592×1944
S6	Apple	iPhone 5	3264×2448
S7	Motorola	Moto G	2592×1456
S8	Samsung	Galaxy S4	4128×3096
S9	LG	G3	4160×3120
S10	LG	Nexus 5	3264×2448
S11	Sony	Xperia Z3	5248×3936
S12	Samsung	Samsung S3	3264×2448
S13	HTC	One S	3264×2448
S14	LG	Nexus 5	3264×2448
S15	Apple	iPhone 6	3264×2448
S16	Samsung	Galaxy S2	3264×2448
S17	Nokia	Lumia 625	2592×1456
S18	Apple	iPhone 5S	3264×2448

**Table 2:** The lowest and highest image resolution in different datasets.

Dataset	Lowest Resolution	Highest Resolution
$D_{L}^{O}$	960×544	5248×3936
$D_{L}^{G}$	960×544	5248×3936
$D_{L}^{W}$	960×544	1600×1200
$D_{L}^{\mathrm{FH}}$	960×544	2048×1536
$D_{L}^{T}$	960×544	1280×960
$D_{V}^{O}$	960×720	5248×3936
$D_{V}^{W}$	960×720	1280×960
$D_{V}^{\mathrm{FH}}$	960×720	2048×1536
$D_{V}^{\mathrm{FL}}$	1040×584	1312×984

**Table 3:** ANN’s architecture.

Type	Multi-Layer Perceptron (MLP)
Number of layers	2
Neurons in input layer	$\left\{ \begin{array}{l} 900\mathrm{for}D_{L}\\ 7480\mathrm{for}D_{V} \end{array}\right.$
Neurons in hidden layer	50
Neurons in output layer	$\left\{ \begin{array}{l} 18\mathrm{for}D_{L}\\ 35\mathrm{for}D_{V} \end{array}\right.$
Learning rule	Back Propagation (BP)
Training function	trainscg
Activation function	logsig
Error	Mean Squared Error (MSE)

**Table 4:** Results (%) of resizing versus cropping the RNs in *original-by-original SI* on $D_{L}^{O}$, by testing different image resolution.

	Resizing					Cropping *				
Size	𝓢𝓔	𝓢𝓟	𝓐𝓡𝓘	𝓕	𝓟	𝓢𝓔	𝓢𝓟	𝓐𝓡𝓘	𝓕	𝓟
1536×1536	0.91	0.99	0.88	0.88	0.95	——	——	——	——	——
1280×1024	0.89	0.99	0.85	0.86	0.94	——	——	——	——	——
1024×1024	0.91	0.99	0.90	0.91	0.96	——	——	——	——	——
960×544	0.90	0.99	0.87	0.88	0.95	0.91	0.99	0.89	0.90	0.95
512×512	0.90	0.99	0.87	0.88	0.94	0.85	0.98	0.81	0.82	0.89
256×256	0.58	0.97	0.55	0.57	0.75	0.76	0.98	0.74	0.75	0.87
128×128	0.18	0.94	0.12	0.17	0.37	0.43	0.96	0.39	0.42	0.66

^*^ The highest resolution for cropping RNs is 960 × 544 px, based on Table 2.

**Table 5:** Results (%) of *original-by-original SI* on different datasets.

Dataset	𝓢𝓔	𝓢𝓟	𝓐𝓡𝓘	𝓕	𝓟
$D_{L}^{O}$	0.91	0.99	0.90	0.91	0.96
$D_{V}^{O}$	0.84	0.99	0.84	0.85	0.894

**Table 6:** Results (%) of *social-by-original SI* on different datasets.

Dataset	𝓢𝓔	𝓢𝓟	𝓐𝓡𝓘	𝓕	𝓟
$D_{L}^{G}-D_{L}^{O}$	0.92	0.99	0.91	0.91	0.97
$D_{L}^{W}-D_{L}^{O}$	0.85	0.99	0.82	0.83	0.92
$D_{L}^{\mathrm{FH}}-D_{L}^{O}$	0.85	0.99	0.82	0.83	0.92
$D_{L}^{T}-D_{L}^{O}$	0.86	0.99	0.83	0.84	0.93
$D_{V}^{W}-D_{V}^{O}$	0.81	0.99	0.79	0.80	0.91
$D_{V}^{\mathrm{FH}}-D_{V}^{O}$	0.80	0.99	0.77	0.77	0.90
$D_{V}^{\mathrm{FL}}-D_{V}^{O}$	0.78	0.99	0.75	0.75	0.89

**Table 7:** Results (%) of *intra-layer UPL* on different datasets.

Dataset	𝓓${}_{L}^{G}$	𝓓${}_{L}^{W}$	𝓓${}_{L}^{\mathrm{FH}}$	𝓓${}_{L}^{T}$	𝓓${}_{V}^{W}$	𝓓 ${}_{V}^{\mathrm{FH}}$	𝓓${}_{V}^{\mathrm{FL}}$
SE	0.91	0.87	0.88	0.87	0.75	0.73	0.43
SP	0.99	0.98	0.99	0.99	0.99	0.99	0.98
ARI	0.88	0.84	0.86	0.86	0.74	0.71	0.40
F	0.89	0.86	0.85	0.85	0.75	0.71	0.42
P	0.96	0.94	0.93	0.92	0.84	0.80	0.58

**Table 8:** Results (%) of *inter-layer UPL* on $D_{L}$.

Dataset	𝓢𝓔	𝓢𝓟	𝓐𝓡𝓘	𝓕	𝓟
$D_{L}^{W}-D_{L}^{G}$	0.90	0.99	0.87	0.88	0.96
$D_{L}^{\mathrm{FH}}-D_{L}^{G}$	0.90	0.99	0.87	0.87	0.95
$D_{L}^{T}-D_{L}^{G}$	0.92	0.99	0.90	0.91	0.96
$D_{L}^{G}-D_{L}^{W}$	0.91	0.99	0.90	0.90	0.96
$D_{L}^{\mathrm{FH}}-D_{L}^{W}$	0.86	0.99	0.83	0.83	0.94
$D_{L}^{T}-D_{L}^{W}$	0.90	0.99	0.88	0.87	0.95
$D_{L}^{G}-D_{L}^{\mathrm{FH}}$	0.90	0.99	0.88	0.88	0.95
$D_{L}^{W}-D_{L}^{\mathrm{FH}}$	0.86	0.98	0.82	0.83	0.94
$D_{L}^{T}-D_{L}^{\mathrm{FH}}$	0.87	0.99	0.84	0.85	0.93
$D_{L}^{G}-D_{L}^{T}$	0.90	0.99	0.88	0.90	0.95
$D_{L}^{W}-D_{L}^{T}$	0.87	0.98	0.85	0.85	0.94
$D_{L}^{\mathrm{FH}}-D_{L}^{T}$	0.87	0.98	0.85	0.86	0.94

**Table 9:** Results (%) of *inter-layer UPL* on $D_{V}$.

Dataset	𝓢𝓔	𝓢𝓟	𝓐𝓡𝓘	𝓕	𝓟
$D_{V}^{\mathrm{FH}}-D_{V}^{W}$	0.80	0.99	0.78	0.79	0.90
$D_{V}^{\mathrm{FL}}-D_{V}^{W}$	0.80	0.99	0.78	0.78	0.88
$D_{V}^{W}-D_{V}^{\mathrm{FH}}$	0.78	0.99	0.76	0.77	0.87
$D_{V}^{\mathrm{FL}}-D_{V}^{\mathrm{FH}}$	0.77	0.99	0.76	0.76	0.87
$D_{V}^{W}-D_{V}^{\mathrm{FL}}$	0.61	0.99	0.58	0.59	0.72
$D_{V}^{\mathrm{FH}}-D_{V}^{\mathrm{FL}}$	0.61	0.99	0.59	0.60	0.73

## Data Availability

The dataset is available from: http://smartdata.cs.unibo.it/datasets##images, accessed on 10 February 2021.
